# Cyanobacteria-Pesticide Interactions and Their Implications for Sustainable Rice Agroecosystems

**DOI:** 10.1155/ijm/7265036

**Published:** 2025-04-01

**Authors:** Sadhana Yadav, Rupanshee Srivastava, Nidhi Singh, Tripti Kanda, Ekta Verma, Piyush Choudhary, Shivam Yadav, Neelam Atri

**Affiliations:** ^1^Department of Botany, Mahila Mahavidyalaya, Banaras Hindu University, Varanasi, Uttar Pradesh, India; ^2^Department of Botany, Magadh University, Bodhgaya, Bihar, India; ^3^Oil and Natural Gas Corporation Ltd., Ministry of Petroleum & Natural Gas, New Delhi, India; ^4^Department of Botany, University of Allahabad, Prayagraj, Uttar Pradesh, India

**Keywords:** biodegradation, cyanobacteria, pesticide, rice, sustainable agriculture

## Abstract

Modern agricultural practices rely heavily on fertilizers and pesticides to boost crop yields, essential for feeding the growing global population. However, their extensive use poses significant environmental risks. Chemical-based fertilizers and pesticides persist in ecosystems, potentially harming ecological stability. Wetland rice farming utilizing nitrogen-fixing cyanobacteria has emerged as an ecofriendly alternative, drawing attention due to its capacity to mitigate pesticide-related issues. Cyanobacteria, capable of fixing atmospheric nitrogen, thrive in low-nitrogen conditions and can aid plant growth. Some species can also biodegrade pesticides, offering a means to clean up contaminated environments. Researchers are exploring ways to leverage cyanobacteria's nitrogen fixation and biodegradation abilities for ecofriendly biofertilizers and environmental cleanup. This approach presents promise for sustainable agriculture and environmental preservation. The current study delves into multiple studies to investigate global pesticide usage levels, primary categorization, and persistence patterns. It also investigates cyanobacterial distribution and their interactions with pesticides in wetland rice ecosystems, aiming to enable their use in sustainable agriculture. Additionally, the review provides a thorough summary of the literature's findings about the potential of cyanobacteria in pesticide degradation.

## 1. Introduction

**Figure 1:**
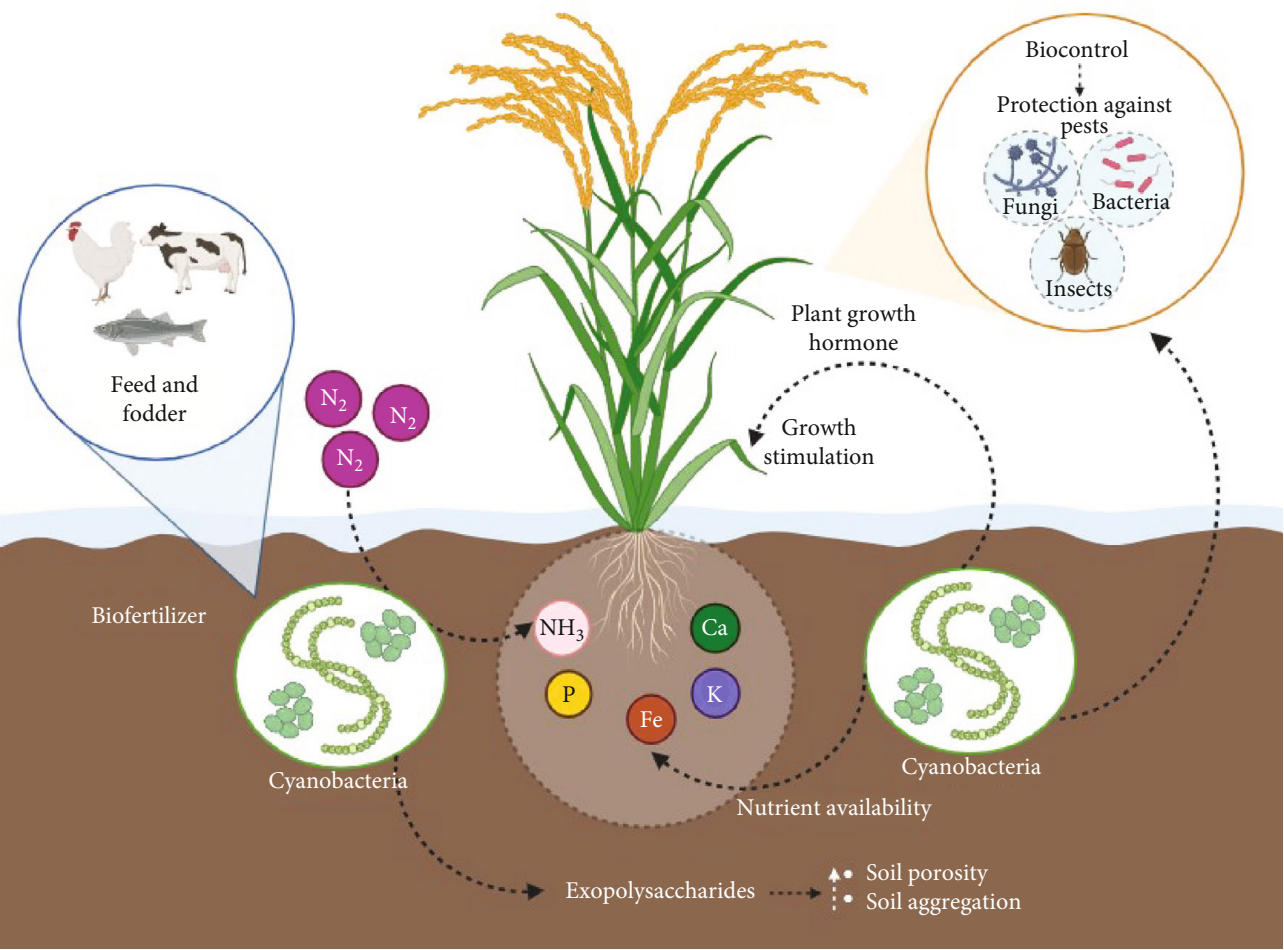
A theoretical illustration of the potential roles of cyanobacteria in ecofriendly and sustainable agriculture.

Cyanobacteria, a group of gram-negative prokaryotic microorganisms, are able to thrive with minimum light, carbon dioxide (CO_2_), or water requirements [1, 2]. They are naturally found from Antarctica to the Arctic poles in a variety of agroecosystems, including paddy fields. They are the earliest known oxygenic photosynthetic microbes on Earth and, during the past three billion years, have significantly contributed to the generation of oxygen in the Earth's atmosphere. Cyanobacteria show promise as microorganisms for sustainable agriculture [3, 4]. A theoretical illustration of the potential roles of cyanobacteria in environmentally friendly and sustainable agriculture is depicted in Figure 1. Scientists and academics are considering the possibility of using cyanobacteria on an industrial scale globally because of their many unique characteristics. Their potential applications include (1) as a resource for biofuel production, (2) as a source of coproducts, (3) nutrition/food supplement, (4) biomolecules and polyunsaturated fatty acids (PUFAs), (5) medicine, and (6) biofertilizers [4–7].

N_2_-fixing cyanobacteria, known as diazotrophs, are cyanobacteria that are helpful in producing inexpensive, readily available biofertilizers that are friendly to the environment. They have the ability to regulate plant nitrogen deficit, enhance soil aeration, increase water-holding capacity, and supplement with vitamin B12 [8, 9]. Within rice crop cultivation regions, several cyanobacteria species have demonstrated highly effective nitrogen fixation abilities. These include *Nostoc linckia*, *Anabaena variabilis*, *Aulosira fertilissima*, *Calothrix* spp., *Tolypothrix* spp., and *Scytonema* spp. [10]. *Anabaena* and *Nostoc* fix up to 20–25 kg/ha of atmospheric nitrogen when living on soil and rock surfaces. However, the use of pesticides in agricultural practices has continuously increased to meet the rising demand for food products [11]. The excessive usage of pesticides and chemical fertilizers poses a major threat to both human health and the environment, as well as significantly polluting both terrestrial and aquatic ecosystems [12–14]. The overuse of synthetic fertilizers increases crop production, but on the other hand, they degrade soil and water quality, increase plant susceptibility to disease, and lower soil fertility [15–19].

Rice is the major staple food of Asian countries, with 505 million tons of annual worldwide production [20]. In Asia, more than two billion people obtain 80% of their energy from rice, which contains carbohydrates (80%), protein (7%–8%), fat (3%), and fiber (3%) [21]. Approximately 87% of global rice production occurs within 11 Asian nations. Around 35% of international rice exports originate from eight of these countries. Rice cultivation across Asia, notably in China and India, significantly influences global food security. Collectively, the sizable economies of China and India account for 49% of worldwide rice yield and are home to 37% of the international population [22]. India had around 46 million hectares of land available for rice farming by the end of the fiscal year 2022, where rice is produced on around 45 million hectares of cultivable land using a massive amount of chemical fertilizer [23]. With the advancement of modern agriculture, the use of herbicides in agricultural production, particularly in paddy fields, has been a regular practice to enhance productivity. Despite having a low toxicity and a short half-life, these types of herbicides are frequently utilized in agricultural production, and as a result, they are “persistent” in the environment. It is a known fact that the increased use of herbicides in recent years has contributed to environmental degradation because these chemicals enter aquatic ecosystems and have detrimental impacts on the entire aquatic environment [24]. One of the biggest threats to public health is the use of pesticides in the food supply. Several studies have examined the pesticide residues found in rice grains [25]. To ensure the safety of the food being consumed, it is crucial to be aware of pesticide residues in both fresh and industrialised food. For instance, OCPs (organochlorine pesticides) are used extensively, particularly in the cultivation of rice paddies because they can boost productivity [26], but they can bioaccumulate in food crops and animal tissues and are hazardous and persistent chemicals [27]. The current situation of the world's population growth in relation to the acceleration of food demand necessitates more paddy cultivation, which involves sustainable pest management through the cautious use of agrochemicals. Sustainable agriculture has gained interest due to its support for low-input soil fertility management and ecosystem health. This counters the negative consequences of conventional agriculture by maintaining nutrient balance and biodiversity.

Sustainable farming practices include soil, water, and managing pests such as crop selection, organic improvements, digging protection, recycling crop residue, soil preservation, soil processing, soil fertility restoration, soil superiority maintenance, biological management of plant diseases, and use of cyanobacteria as potential biofertilizers, biostimulants, and biopesticides. The soil is an essential part of sustainable agriculture since it is the primary source of crop manufacture and a composite reservoir of life that requires nutrients for long-term productivity and constancy [28]. The biological soil crust (BSC), which is the topmost layer of the soil and is inhabited by a variety of microorganisms including cyanobacteria, microalgae, actinomycetes, fungi, and bacteria, is particularly conducive to their growth and is crucial for enhancing agricultural productivity and soil fertility [29]. These “beneficial microbes” offer a tremendous opportunity for sustainable and environmentally friendly agricultural practices such as recycling of nutrients, breakdown of organic waste, detoxification of toxic chemicals, management of plant pathogens, and production of metabolites such as vitamins, hormones, and enzymes [19, 30]. Due to their high organic content, which preserves the soil's ability to store water and sustain the availability of minerals while enhancing soil fertility and physicochemical qualities, cyanobacterial fertilizers are also thought to be superior to chemical and farmyard manure fertilizers [31, 32]. Hence, cyanobacteria have been viewed as a possible alternative for minimizing the usage of agrochemicals and their negative effects. This review is aimed at critically analyzing the effects of pesticides and their residues in rice paddy fields. A thorough discussion is provided on the role of cyanobacteria in agriculture and their potential use to biodegrade pesticides. Additionally, the development of bacterial-microalgal consortia is explored for efficient biodegradation of pesticides through synergistic degradation pathways. The ability to harness natural biodegradation processes has implications for sustainable pesticide management and reduced ecological impacts in agroecosystems.

## 2. Types of Pesticides

**Figure 2:**
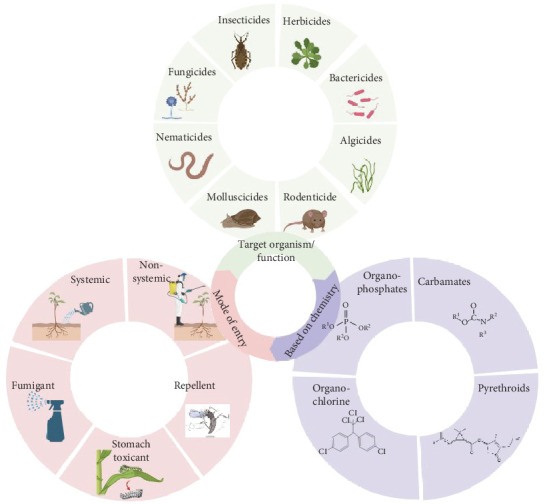
Major classification of pesticides as proposed by Drum [33].

Pesticides are chemical compounds used to eliminate or deter pests across a variety of applications. Broadly defined, pesticides include insecticides, herbicides, fungicides, rodenticides, garden products, household disinfectants, and wood preservatives. While sharing a common function, these substances exhibit diverse physical, chemical, and structural properties depending on the target pest. A scientifically rigorous classification system is essential to categorize pesticides based on their distinguishing characteristics. At present, there are three major bases for pesticide classification as proposed by Drum [33] (Figure 2). These three widely used approaches to categorize pesticides include (i) classification based on pesticide function and targeted pest organism, (ii) classification according to entry mechanism, and (iii) classification derived from pesticide chemical composition.

### 2.1. Classification Based on Pesticide Function and Targeted Pest

Pesticides can be classified based on the taxonomic group of the target pest. Compound names aim to reflect activity by using a suffix derived from the Latin root “cide,” meaning to kill, after the name of the pest controlled. Examples include insecticides for insects, herbicides for plants, and fungicides for fungi. Not all pesticide names use the “cide” suffix. Classification based on pesticide function and targeted pest organism is listed in Table 1.

### 2.2. Classification According to Mechanism of Entry

The modes by which pesticides interact with or penetrate target organisms are referred to as modes of entry. Five primary pesticide modes of entry include systemic, contact, stomach poisons, fumigants, and repellents. Systemic pesticides enter an organism's vascular system, typically through roots or foliage, and are translocated through the xylem and phloem to other plant parts. Contact pesticides must physically contact the pest to be effective and are often applied as sprays. They control pests on contact but do not persist on the treated surface or within the plant. Stomach poisons are orally ingested by pests and act upon the digestive system. Fumigants are pesticides applied as gasses or vapors that penetrate enclosed spaces through the air to control pests inhabiting that space. Lastly, repellents deter pests through olfactory or contact stimuli and prevent infestations from occurring rather than causing direct mortality like other modes of entry. Understanding a pesticide's mode of entry is integral to determining its effectiveness against various types of pests and selecting appropriate application methods.

### 2.3. Classification of Pesticide Based on Pesticide Chemical Composition

Pesticides are classified based on their active ingredients' chemical composition and nature. This classification method provides insight into the efficacy, physical properties, and chemical properties of different pesticides. Knowledge of pesticides' chemical and physical traits informs appropriate application mode, safety during use, and application rates. Based on composition, pesticides comprise four main groups: organochlorines, organophosphates, carbamates, and pyrethrins and pyrethroids. This classification provides a scientifically sound method for understanding and utilizing pest control agents in a safe and effective manner. Classification based on pesticide chemical composition is listed in Table 1.

## 3. Mechanism of Action of Pesticides

**Figure 3:**
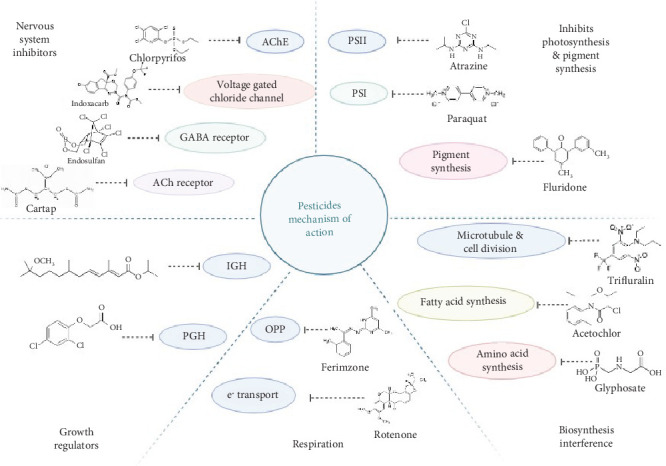
Mechanism of action of certain commonly used pesticides. AChE, acetylcholinesterase; GABA, gamma-aminobutyric acid; IGH, insect growth hormone; PGH, plant growth hormone.

Currently, agricultural pests are primarily controlled through the application of approximately 715 distinct chemical pesticides (United States Environmental Protection Agency, 2022). These pesticides are estimated to act via around 95 different mechanisms to disrupt pest behaviors, physiology, or reproduction [34]. Some excellent reviews discussing the mechanism of action of pesticides in detail are by Casida [35, 36].  Figure 3 displays the mechanism of action of certain commonly used pesticides.

The majority of contemporary pesticides function by targeting the nervous system of insect pests, with some of the major modes of action including acetylcholinesterase inhibition, gamma-aminobutyric acid (GABA)–gated chloride channel antagonism, and sodium channel modulation [37, 38]. Other common mechanisms encompass lipid biosynthesis inhibition, mitochondrial disruption, and hormone mimicry [34, 39]. Common targets and associated mechanisms of action are discussed here.

Insecticides primarily act on the nervous system at synapses or axons. The principal target is the cholinergic system, with organophosphates and methylcarbamates inhibiting AChE to prolong acetylcholine action. Neonicotinoids act as competitive agonists for acetylcholine at nicotinic receptors. Spinosyns act as allosteric modulators of nicotinic acetylcholine receptors. Cartap is a noncompetitive antagonist of the same target. The voltage-gated sodium channel located in axons is modulated by DDT, pyrethrins, and pyrethroids. Oxadiazines and semicarbazones block this channel. The GABA-gated chloride channel involved in synaptic transmission is targeted by noncompetitive antagonists/blockers from two classes—cyclodienes and phenyl pyrazoles. Avermectins stimulate the glutamate-gated chloride channel. The G-protein-coupled octopamine receptor is agonized by amitraz. Newly introduced diamides targeting ryanodine receptors offer promise for lepidopterous larvae based on potency and safety.

Herbicides that disrupt plant-specific processes are less toxic to mammals as they lack similar targets. Photosystem II is an early herbicide target and remains important, with around 50 compounds acting on it. Bipyridilium herbicides like paraquat divert the PSI electron flow. Also, protoporphyrinogen IX oxidase is targeted by 26 herbicides from different classes, producing singlet oxygen and membrane damage. Phytoene desaturase is very sensitive to some herbicides with m-trifluoromethylphenyl groups.

Another target of a different class of pesticide is the biosynthesis of essential biomolecules. Amino acid biosynthesis within plants represents a significant target for herbicide inhibition, as plants possess the innate ability to synthesize their own amino acids de novo. The three primary herbicide targets involved in amino acid metabolism include enolpyruvylshikimate-3-phosphate (EPSP) synthase, acetohydroxy acid synthase (AHAS), and glutamine synthase. Inhibition of these enzymes disrupts critical amino acid biosynthesis pathways, conferring potent herbicidal effects. Apart from amino acid biosynthesis, microtubule synthesis/organization and fatty acid biosynthesis are other important targets for herbicides. Various fungicides and insecticides also have important targets from the biosynthetic pathways of important biomolecules such as sterol, nucleic acid, protein, lipid, and glucans.

Respiration is another important target for insecticides, herbicides, and fungicides. Pesticides can disrupt mitochondrial function through several mechanisms. Binding at important sites may inhibit oxidative phosphorylation and prevent the formation of the proton gradient needed for ATP production. Uncoupling prevents phosphorylation by disrupting the proton gradient.

Another important target for certain pesticides is delicate hormone processes in insects, plants, and fungi. Insect growth regulators (IGRs) and plant growth regulators (PGRs) regulate insect growth and plant growth, respectively. Regulators of fungal disease development or host plant defense inducers have the ability to modulate fungal disease progression or trigger host plant defensive responses. IGRs mimic or block the actions of juvenile hormones and related compounds that direct an insect's development, including molting and metamorphosis from immature stages to reproductive adulthood. By preventing an insect from developing properly, IGRs disrupt the lifecycle and inhibit populations from sustaining themselves. PGRs function similarly to manipulate the growth and form of agricultural crops.

## 4. Trends Regarding Worldwide Pesticide Consumption

**Figure 4:**
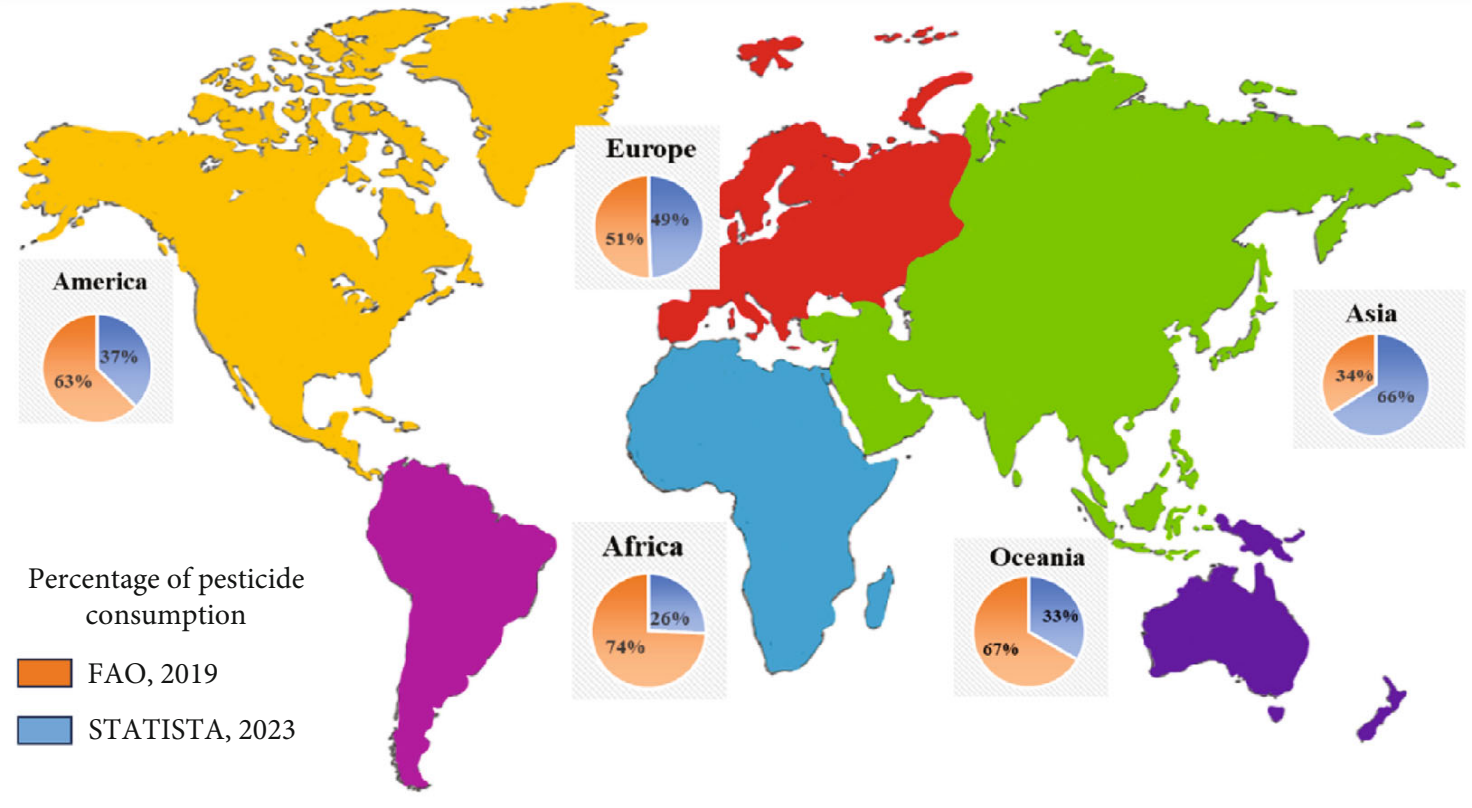
Percent consumption of pesticides according to FAO (2019) and Statista (2023).

In modern agriculture, pesticides are widely consumed and are an efficient and affordable approach to increase production capacity and quality, assuring the security of food for the world's ever-increasing population. According to the WHO, by 2050, the population of the world is anticipated to grow from 7.7 billion to 9.7 billion [40]. In a continually increasing population, feeding the world's expanding population will be impossible without a substantial rise in agricultural production. More than half of the world's estimated 2 million tons of pesticides are used in Asian countries, with the Americas and Europe coming in second and third. A review of global pesticide usage statistics from 1990 to 2018 indicates that consumption patterns vary significantly by continent. Asia represented the largest consumer of pesticides, accounting for approximately 53% of total global usage over this time period. North and South American countries, collectively categorized as the continent of America, constituted the second highest pesticide-utilizing region at 30%. Europe followed with 14% of overall international pesticide consumption. Despite the fact that agriculture is a major source of income for most African countries, only a small portion of the continent's total pesticide consumption, that is, around 2%, is consumed by Africa. The remaining 1% of global pesticide usage is contributed by Oceania. Based on statistics from the FAO, the data on the average global distribution of pesticide consumption ranged from 1990 to 2018, as shown in Figure 4.

**Figure 5:**
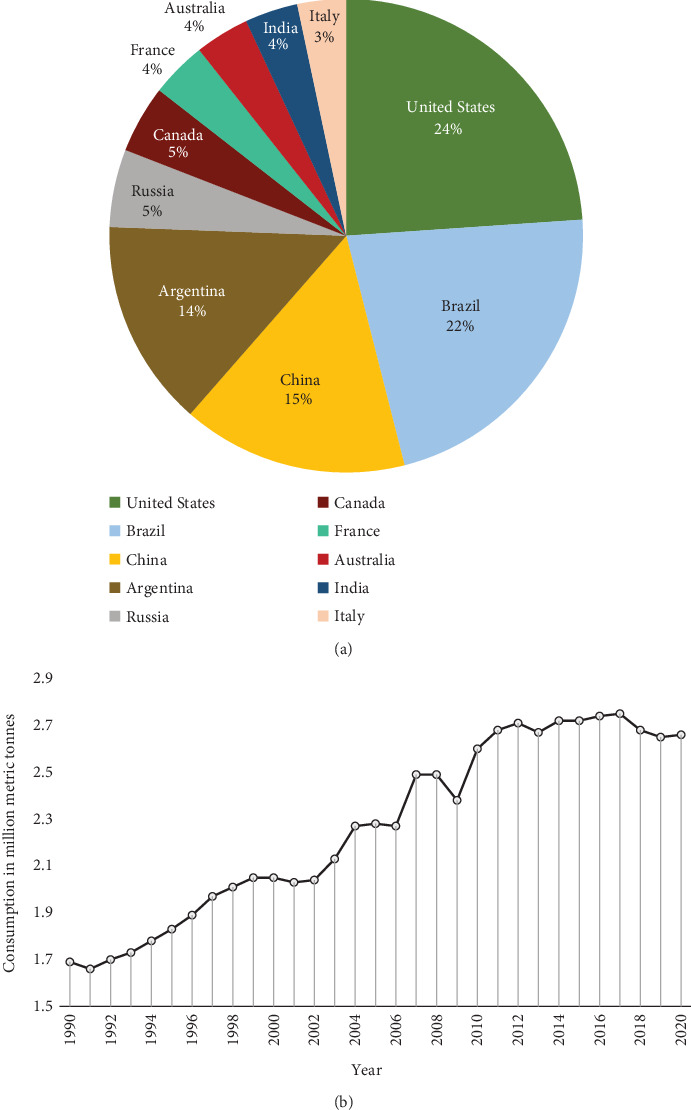
(a) Pesticide usage consumption in different countries across the globe. (b) Consumption (million metric tons) from 1990–2020 [41].

**Figure 6:**
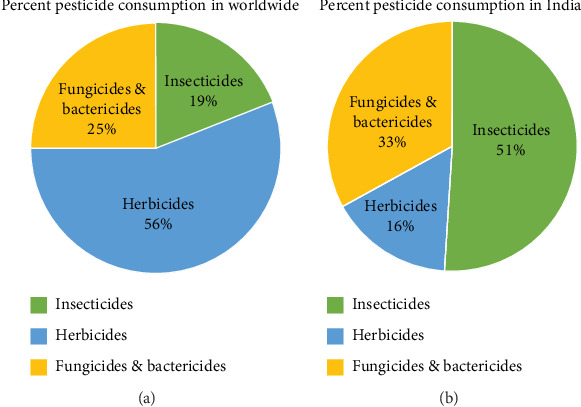
Worldwide and Indian pesticide usage patterns [41, 42].

The Top 10 pesticide-consuming nations globally in 2020 were the United States, Brazil, China, Argentina, Russia, Canada, France, Australia, India, and Italy. As shown in Figure 5a, the United States consumed the highest quantity of pesticides that year at approximately 407,800 metric tons. Brazil consumed the second-highest amount at 377,200 metric tons. Global pesticide consumption in 2020 was estimated to be 2.6 million metric tons. While consumption has fluctuated somewhat in the past decade, it has remained relatively consistent, declining only slightly from 2.8 million metric tons in 2011 to the reported 2.6 million metric tons in 2020 (Figure 5b). Increasing demand for crop protection chemicals reflects the need to enhance agricultural productivity and food security given climate change, pest resistance, and population growth challenges. A recent global survey of pesticide usage reported that approximately 56% of total pesticides, by mass, were herbicides [41]. Fungicides and bactericides collectively comprised approximately 25% of the total, while insecticides represented about 19% of pesticides used worldwide (Figure 6).

Insecticide consumption accounted for 51% of total pesticide use in India in 2021, followed by 33% for fungicides and bactericides and 16% for herbicides, as depicted in Figure 5 [41]. In contrast to global trends, pesticide application in India faces challenges from the use of low-quality pesticides and a lack of information regarding proper pesticide usage. Insufficient regulation of pesticide usage has resulted in increased detection of pesticide residues in Indian foodstuffs, according to the economic survey of 2015–2016 [43, 44]. Figure 6 illustrates global and Indian pesticide usage patterns [41, 42].

India has registered 293 pesticides and continues the production or usage of 104 pesticides prohibited in two or more other countries [41]. Currently, India ranks as the second largest pesticide producer in Asia and 12th globally in terms of pesticide consumption ([45, 46]; FAOSTAT). Also, India ranks fourth worldwide in pesticide production. In India, cotton pest management alone utilizes 50% of total insecticide usage [41]. Insecticides are applied more frequently than herbicides or fungicides, with crops such as cotton, wheat, and rice demanding significant pesticide quantities for management [47].

**Figure 7:**
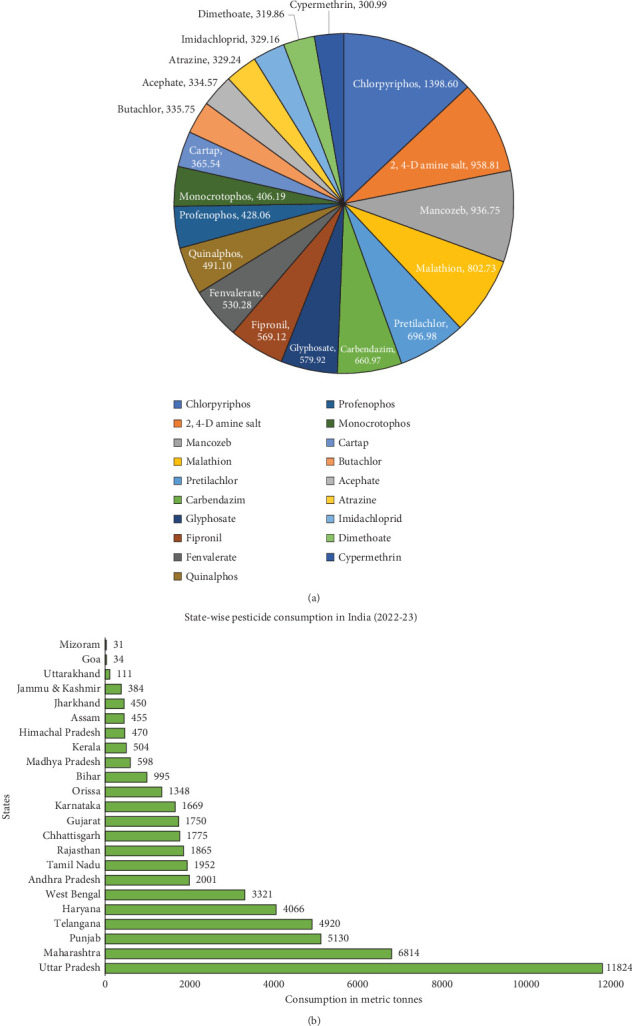
(a) Most consumed pesticides in Indian agriculture in 2022–2023. (b) State-wise consumption in 2022–2023 (Statistical Database, Directorate of Plant Protection, Quarantine & Storage).

Among different states, in 2022–2023, Uttar Pradesh reported the highest pesticide consumption nationwide, followed by Maharashtra, Punjab, and Telangana (Figure 7). Punjab utilized pesticides at the highest application rate of 0.74 kg/acre, followed by Haryana (0.62 kg) and Maharashtra (0.57 kg). Available data indicates that 41% of all pesticides used in India are applied in Maharashtra and Uttar Pradesh. The most commonly used pesticide is chlorpyrifos, with 1398.6 metric tons usage, followed by 2,4-D, mancozeb, and malathion (Figure 7a). Consumption has sharply decreased in states such as West Bengal, Gujarat, and Karnataka while rising in Kerala and Chhattisgarh, as shown in Figure 7b. The Top 6 pesticide-consuming states in India account for over 70% of national crop pesticide usage [48]. Common herbicides applied in Indian rice fields pre-emergence include pretilachlor, butachlor, metsulfuron-methyl, and pendimethalin, while postemergence herbicides include atrazine, anilofos, ethoxysulfuron, bispyribac sodium, 2,4-D, glyphosate, diquat, and paraquat.

## 5. Colonization of Cyanobacteria in Paddy Field

Cyanobacteria, also known as blue-green algae, comprise a diverse and highly successful group of photosynthetic prokaryotes that were among the earliest forms of life to evolve oxygenic photosynthesis. Cyanobacteria have persisted on Earth for billions of years and remain ubiquitous across aquatic and terrestrial environments. Taxonomically, cyanobacteria consist of approximately 150 genera and over 2000 known species exhibiting remarkable morphological diversity, ranging from unicellular and colonial forms to complex filamentous and branched filamentous morphologies. Five major subsections are delineated based on structural characteristics: Chroococcales, Pleurocapsales, Oscillatoriales, Nostocales, and Stigonematales [49].

This wide range of morphologies reflects cyanobacteria's extraordinary physiological adaptability and tolerance to varying environmental conditions. Cyanobacteria can thrive across a broad spectrum of temperatures, salinities, water potentials, pH levels, and light intensities. Their pervasive distribution on Earth demonstrates highly developed mechanisms for coping with different environmental stresses. From an applied perspective, cyanobacteria are economically important as biofertilizers in agriculture due to their nitrogen-fixing capabilities. Many species are able to grow successfully in habitats with little to no available fixed nitrogen by converting atmospheric nitrogen gas into bioavailable forms via nitrogen fixation. This process occurs in specialized cells termed heterocysts.

Since ancient times, paddy fields have traditionally been grown in bigger, lowland tropical regions that are favorable to the development of diazotrophic, oxygenic cyanobacteria by supplying the ideal temperature, vital nutrients, and water resources [50, 51].

**Figure 8:**
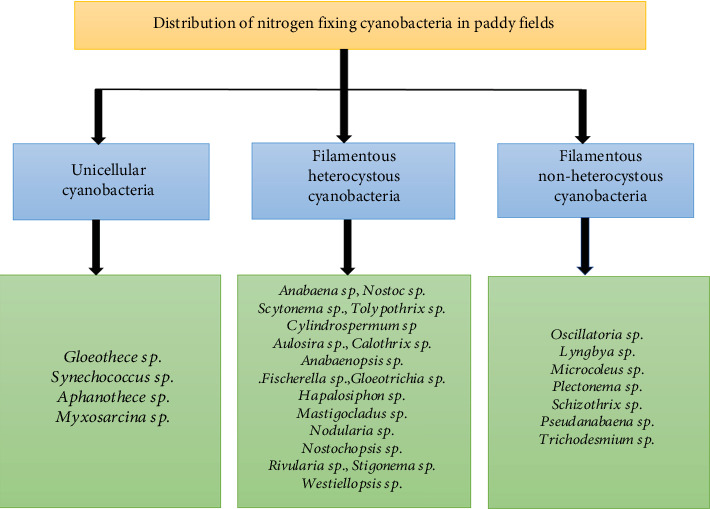
Biodiversity of cyanobacteria in Indian paddy fields.

Based on available data, cyanobacteria constitute approximately 70% of the algal flora observed in Indian rice paddy fields. Potential diazotrophic cyanobacteria genera identified include *Anabaena*, *Nostoc*, *Calothrix*, *Aulosira*, *Anabaenopsis*, *Tolypothrix*, *Cylindrospermum*, *Hapalosiphon*, and *Fischerella* (Figure 8). The most prevalent cyanobacterium among them is *Aulosira fertilissima*, which is also recognized as the most potent nitrogen-fixer [52]. The diazotrophic filamentous cyanobacteria, especially *Nostoc* spp. and *Anabaena* spp., are widely dispersed in wetland rice habitats and maintain field fertility through the fixation of atmospheric nitrogen [53].

Several studies have investigated cyanobacteria species in rice paddy fields across India. Prof. R. N. Singh [54–56] identified *Aulosira fertilissima*, *Anabaena ambigua*, and *Cylindrespermum gorakhperense* as the most common species. Saadatnia and Riahi [57] found *Anabaena* to be significant occupants of rice rhizospheres, unlike Prasanna and Nayak [23], who reported *Nostoc* and *Anabaena* as the most prevalent genera. Singh [49] also isolated numerous heterocystous cyanobacteria across India, including *Anabaena*, *Nostoc*, *Cylindrospermum*, *Hapalosiphon*, *Mastigocladus*, and *Fischerella*. *Scytonema* and *Calothrix* were identified in 50% and 15% of rice soils, respectively, while *Nostoc* and *Anabaena* were found in nearly all soils outside India, as per Reynaud and Roger [58]. Tiwari et al. [59] discovered seven nonheterocystous filamentous isolates (*Pseudanabaena*, *Phormidium*, *Plectonema*, *Oscillatoria*, *Lyngbya*, *Limnothrix*, and *Microcoleus*) from Uttar Pradesh among 28 species.

## 6. Pesticide Effect on Paddy Field Cyanobacteria

Pesticide application in agroecosystems like rice paddies can initially benefit pest control, but prolonged use destabilizes the soil microbiome. Specifically, pesticides disturb cyanobacterial communities, significantly altering native ecology and microflora. While a combination of biocides caused an additive effect on filamentous cyanobacteria, it was found to be growth-promoting in the case of unicellular forms [60, 61]. This disrupts nitrogen fixation and physiological processes in rice, impacting growth. Dash et al. [62] observed an adverse impact of the biocides on the nitrogenase activity of cyanobacteria. Prudent pesticide management is needed to maintain soil ecosystem stability and function [63–65]. Kaushik et al. [66] reviewed the impact of these agrochemicals on the rice field cyanobacteria and its implications for the rice crop. The widespread use of pesticides in the agriculture field negatively affects the growth and physiology of cyanobacteria, possibly by producing reactive oxygen species (ROS) [4, 51]. ROS are well documented to cause damage to biological macromolecules, including DNA, RNA, and lipids [67–69]. In another instance, pesticides function as growth promoters, causing some cyanobacterial species to grow excessively and creating a bloom state, which causes anoxygenic conditions to kill off a variety of natural microflora. When pesticides are applied to soil and aquatic ecosystems in small amounts or at the beginning stages, they have a positive effect on soil productivity and microorganism functions. However, when pesticides are applied repeatedly, they become toxic and inhibit various biological processes in the organisms, such as cyanobacteria's biological nitrogen fixation [63]. Kaushik et al. [66] reviewed the impact of these agrochemicals on the rice field cyanobacteria and its implications for the rice crop. Detailed studies on the effects of pesticides like insecticides, herbicides, and fungicides on growth and N_2_-fixation on the soil-dwelling cyanobacteria are presented in Table 2.

In paddy fields, weed growth is a typical occurrence, and its detrimental effect on rice production has been noted globally. Farmers constantly apply large amounts of herbicide on rice fields to prevent weed growth, which has a negative influence on a number of nontarget organisms, the most prevalent of which are cyanobacteria [93]. Anilofos, bentazone, pyrazosulfuron-ethyl, fenoxaprop-P-ethyl, and pyribenzoxim are frequently employed herbicides in rice crop fields. In China, paddy fields are heavily treated with the herbicides penoxsulam, metamifop, bispyribac sodium, and cyhalofop-butyl to eradicate weeds [94].

Butachlor is a pre-emergence herbicide that has been shown to be moderately to highly harmful to cyanobacteria [95]. It was found that *Anabaena*, *Aulosira*, and *Anabaenopsis* are more sensitive to butachlor, whereas cyanobacteria like *Nostoc*, *Calothrix*, *Westiellopsis*, *Tolypothrix*, *Rivularia*, *Fischerella*, *Leptolyngbya*, *Gloeotrichia*, and *Cylindrospermum* seemed to be tolerant against butachlor [96]. Panda et al. [88] performed a comparative performance by LD_50_ dose of methyl viologen (MV)/paraquat on three strains of the nitrogen-fixing, heterocystous, photoautotrophic cyanobacterium *Anabaena. Anabaena* 7120 and *Anabaena* L-31 displayed better tolerance than *Anabaena doliolum.* Proteomic studies revealed the upregulation of proteins involved in oxidative stress alleviation and protein homeostasis upon MV exposure in three strains, with the downregulation of crucial photosynthesis and carbon metabolism enzymes. Similarly, paraquat exposure was found to produce significant dose-dependent toxicity, including a drop in the biomass, Chl-a, and phycocyanin contents in *Nostoc* sp. N1 and *Anabaena* sp. A1 [97]. Another study conducted by Naaz et al. [98] showed dose-dependent paraquat toxicity in rice biofertilizer *Microchaete* sp. NCCU-342. Furthermore, they also made an attempt to enhance paraquat resistance by exogenous addition of salicylic acid. Their study suggested that, for sustainable agricultural practices, especially those involving paddy field cyanobacterial biofertilizers, the application of salicylic acid or organisms with enhanced salicylic acid productivity may serve as a viable option to circumvent issues arising from paraquat toxicity. Monosulfuron is a sulfonylurea herbicide that significantly inhibits growth and in vitro acetolactate synthase activity in diazotrophic cyanobacteria *Aphanizomenon flos-aquae* and *Anabaena azotica.* Based on experimental data, it was proposed that the mechanism of action involves disruption of protein metabolism through inhibition of the biosynthetic pathways responsible for producing branched-chain amino acids [84]. Monuron is a nonselective systemic herbicide that inhibits photosynthesis. Sachu et al. [99] studied the effect of monuron on the cyanobacterium *Nostoc muscorum* Meg 1 on photosynthetic pigments, heterocyst frequency, RuBisCO, nitrogenase and GS activities, photosystem II, proteins, and carbohydrate production. Toxicity from monuron exposure was dose-dependent, suggesting inhibition of protein synthesis and degradation of enzymes at higher concentrations. Glyphosate is a broad-spectrum organophosphorus systemic herbicide and crop desiccant. 2,4-D is a common systemic herbicide that controls broadleaf weeds. Tansay et al. [100] studied the impacts of field concentrations of glyphosate and 2,4-D on the cyanobacterium *Nostoc* sp. N1 as well as rice seedlings. Results indicated higher toxicity of glyphosate than 2,4-D. However, both herbicides were found to increase the biomass and chlorophyll a content of *Nostoc* sp. N1, suggesting their potential to stimulate growth and photosynthesis. *Nostoc* sp. N1 cells promoted rice seedling germination alone and alleviated toxicity from two herbicides, suggesting the potential of *Nostoc* sp. N1 cells for mitigating herbicide residue toxicity in rice paddies. Another group analyzed the effect of atrazine on diazotrophic *Cylindrospermum stagnale* and also attempted to reduce atrazine stress by the addition of indole acetic acid (IAA). Atrazine toxicity had a dose-dependent effect on *C. stagnale*, and additional research showed that both exogenous and endogenous IAA counteracted atrazine's negative effects. Further, it reduced the levels of MDA while simultaneously boosting the concentration of chlorophyll and several antioxidant enzyme activities such as SOD, CAT, and APX activity. Therefore, using IAA or cyanobacterial biofertilizer that releases enough IAA may help sustainable agriculture minimize the toxicity of atrazine [86].

Insecticide affects the neurological system of the pest or pathogen, thereby halting their growth. Some of the most popular insecticides utilized in paddy fields around the world include cypermethrin, endosulfan, pyridaphenthion (PY), carbofuran, diazinon, BHC (benzene hexachloride), malathion, and acetamiprid. The greater and continuous use of insecticides in rice paddies and crop systems has demonstrated detrimental effects on cyanobacterial growth, productivity, nitrogen fixation, and protein levels. Among several insecticides, organochlorine insecticides have a significant impact on the cyanobacterial population [101, 102]. The cyanobacterial flora is also negatively impacted by the field application of DDT. According to a study by Hutber et al. [103], *Anabaena variabilis* and *Nostoc* strain MAC growth was significantly inhibited when exposed to a 50–60 ppm DDT solution. Similarly, BHC was tolerated up to 55 ppm by *Cylindrospermum* sp., *Plectonema boryanum*, and *Aulosira fertilissima* [104]. Furthermore, Das and Singh [105] found that BHC at 40 ppm was bacteriostatic to *Anabaenopsis raciborskii*, and at 50 ppm was bactericidal to *Anabaena aphanizomennoides*. However, some BHC variants were also reported to promote *Microcystis aeruginosa* development [106]. Chanu et al. [71] reported that alpha-cypermethrin inhibited the growth and photosynthetic pigments of cyanobacterium strain *Anabaena* sp. NC-K1, while enhancing the oxidative damage and enzymatic antioxidants and proline content at lethal concentration. PY negatively affects the cell growth of *Anabaena laxa* and *N. muscorum* after 7 days of exposure. It significantly decreased the total Chl amount in *N. muscorum* and *A. laxa* by 5% and 25%, respectively [74]. At 25 ppm, carbofuran promoted the growth of *N. muscorum* in paddy fields, but concentrations over 1000 ppm inhibited cyanobacteria [107].

Metalaxyl is an acylalanine systemic fungicide used to control a variety of fungal diseases. Various studies have reported that on paddy, metalaxyl had little effect on the variety of filamentous cyanobacterial species, including *Nostoc* and *Anabaena* spp. [53]. Hamed et al. [92] reported that metalaxyl reduced growth, pigment content, and photosynthetic enzymes such as PEPC and RuBisCo of *Anabaena laxa* and *N. muscorum.* Mancozeb, a nonsystematic dithiocarbamate fungicide extensively utilized in agricultural crops, particularly paddy, is hazardous to cyanobacteria [78, 108]. According to some studies, tebuconazole also suppressed the development of *Anabaena fertilissima*, *Westiellopsis prolifica*, and *Aulosira fertilissima*. It also prevented the synthesis of enzymes such as glutamine synthetase, nitrate reductase, and succinate dehydrogenase and affected carbohydrates and pigments used in photosynthetic processes [102, 109, 110].

## 7. Presence of Pesticide Residues in Rice Crop and Its Impact

Pesticide residues present in the environment have grown to be a significant issue in several countries. Residue concentrations of pesticides in food products are a significant cause for worry due to their chemical characteristics directly affecting human health worldwide. Nowadays, the majority of agricultural foods contain prohibited levels of pesticide residues. Pesticide residues are transmitted from lower to higher trophic levels along the food chain, and this process also causes residues to be biomagnified [111]. Dors et al. [112] examined the distribution of pesticides such as carbofuran, clomazone, bispyribac sodium, and tebuconazole in different fractions of milled rice. They found that only clomazone and tebuconazole were within acceptable limits, while all other pesticides were beyond acceptable limits. The presence of insecticide residues in rice differs based on the kind of grain (hull, polished grain, or bran) and also the functional categories found in pesticides [25]. Immediate solutions are required to assure food safety because pesticide residue in food poses a severe risk to public health [113, 114]. According to Kong et al. [115], the half-lives of metamifop in rice, water, and soil were 2.2–3.5, 1.3–2.3, and 11.7–20.2 days, respectively, whereas the half-life of bispyribac sodium was 3.0–3.8 days in the rice plant, 5.0–5.6 days in the soil, and 1.4–2.2 days in the water sample of the controlled field tests. The limit of quantification (LOQ) for BYS residues was 5.0 g/kg in rice plants, 2.0 g/kg in rice hulls, 0.2 g/kg in water, and 0.1 g/kg in soil and husked rice [116]. BYS is hazardous to nontarget aquatic and terrestrial organisms, with extremely harmful effects reported [117–119]. BYS was detected in irrigation water for rice crops at up to 3.5 *μ*g/L, indicating it may contaminate water through leaching or runoff, given its high water solubility of 64 g/L [120].

The half-life of pendimethalin in transplanted rice (TPR) soil varied from 2.22–2.80 days initially and 23.51–24.66 days. Postharvest soil residues were below detection limits in direct-seeded rice (DSR) as well as TPR. However, at 1.0 and 2.0 kg/ha, DSR residues of 0.005 and 0.007 *μ*g/g and TPR residues of 0.003 and 0.005 *μ*g/g were found in rice grain, respectively. In both the DSR and TPR treatments, rice straw residues were 0.003–0.006 *μ*g/g [121]. Another study analyzed the effect of long-term application of the herbicide pendimethalin in a maize-wheat crop rotation on the persistence of said herbicide [122]. There remained extremely small or negligible amounts of pendimethalin residue in the soil due to gradual disintegration during the rice-growing season and leaching from frequent irrigation water (ranging from 0.02 to 0.07 *μ*g/g or mg/kg). At observed application rates, the half-life of bispyribac sodium in 2016 and 2017 was 4.06–13.93 and 3.19–11.95 days, respectively, after rice was harvested [123]. By using HPLC-MS, pendimethalin was measured, and detection values were linear in the range of 1.0–10.0 *μ*g/L. The LOQ was 2.5 × 10^−4^ g/L for water and 1 × 10^−2^ g/kg for tissues, sediments, and waterweeds. Recoveries ranged from 86.9% to 103.5%. In Hubei Province from 2018 to 2020, pendimethalin was found in 8.67% of samples, with its concentrations ranging from 1.95 to 8.26 *μ*g/kg [124].

At 84 and 126 g a.i./ha, the residual oxadiargyl concentrations in rice straw, rice hull, paddy water, brown rice, and soil all stayed below the LOQ (20 g/kg) [125]. The half-lives (t^1/2^) of oxadiargyl in paddy soil ranged from 4.5 to 7.6 days, with a dissipation curve fitting to a first-order kinetic equation for paddy soil. The final oxadiargyl residues were undetectable in rice straw. There are maximum residue limits (MRLs) for oxadiargyl in rice, such as 0.01, 0.05, and 0.02 mg/kg in the European Union, Japan, and China, respectively [125, 126]. In research, 351 of crops in nations other than China, 10 pyrethroids (cypermethrin, permethrin, bifenthrin, deltamethrin, fenvalerate, 350-cyhalothrin, fenpropathrin, cyfluthrin, allethrin, and tetramethrin) at concentrations ranging from 3.00 to 573.00 ng/g deltamethrin and cypermethrin were often found, and the amounts of cypermethrin were significantly higher than those of other pyrethroids. In the lake of Savar, diazinon was found at a concentration of 7.86 *μ*g/L [127] and in the Manikganj paddy field at a concentration of 0.027 g/L [128]. According to Chowdhury et al. [129], carbaryl was the most frequent pesticide found in Dhamrai Upazila at 14.1 and 18.1 g/L, whereas carbofuran was found to be contaminated at 105.2 g L^−1^in another water sample from the same area.

The topsoil from a tropical riverbank basin's rice–vegetable rotation was tested for pesticide residues. The 256 tropical topsoil samples were collected between 2018 and 2019 in China's Hainan province's Nandu River Basin (NRB) and Wanquan River Basin (WRB). A process based on a QuEChERS methodology was used to find a total of 32 current-use pesticides (CUPs) and nine legacy pesticides (LPs). The insecticide imidacloprid (IMI) (139.4 g/kg) and the fungicide carbendazim (257.2 g/kg) had the highest residual levels. These results demonstrated that residues of IMI ranging from 7.0 g/kg to 1.9 mg/kg were present in agricultural soils. Numerous studies have been conducted on the DDT and HCH contamination of tropical agricultural soil. The average concentration of HCHs and DDTs was 3.5 g/kg, with mean concentrations ranging from 0.81 to 14.9 g/kg [130]. All the above data on pesticides, their half-lives, and detected residues are shown in Table 3.

**Figure 9:**
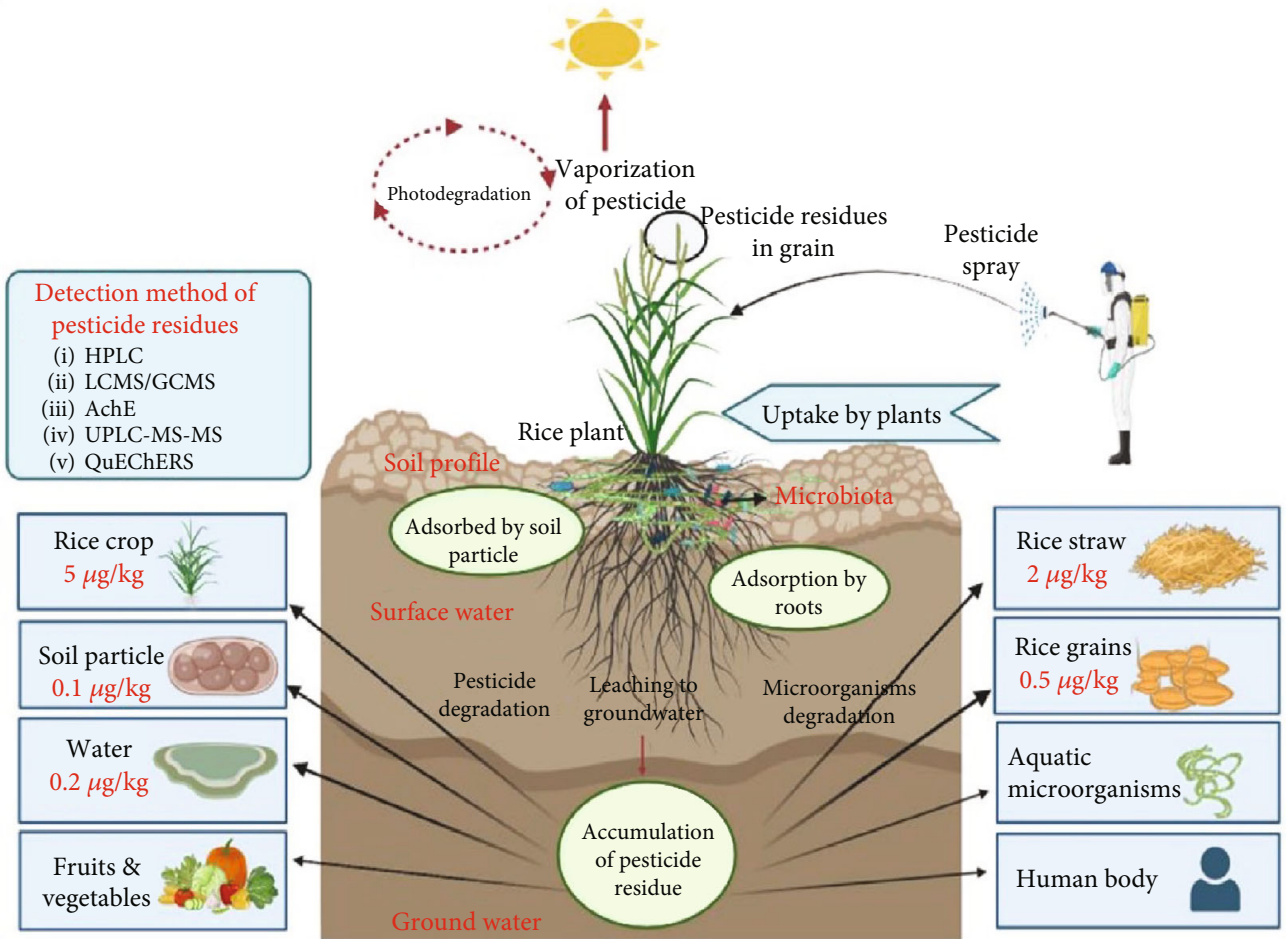
Impact of pesticide residue on food, rice crop, and microbiota of soil.

Two new sensitive and selective UHPLC-MS/MS methods were developed to quantify pesticide residues in paddy soil and water samples, as shown in Figure 9. The methods demonstrated high sensitivity and selectivity by quantifying concentrations as low as 0.1 ng/mL in water and 0.5 ng/g in soil, showing the capability to detect trace amounts. Isoprothiolane showed the highest concentration (34.81 ng/g) in paddy soil. Chlorantraniliprole showed the highest concentration in the paddy water sample. See Figure 9 for data (11.83 ng/mL) [135]. In India, butachlor is a commonly used granule-based postemergence herbicide on rice.

The fertility of the soil and the quantity of soil organisms are declining as a result of the continued use of these synthetic compounds. Butachlor has a half-life of 1.65–2.48 days in field water and 2.67–5.33 days in soil [136]. The voltametric technique was developed to find butachlor in soil samples. There was an excellent linear response to butachlor at concentrations between 0.10 and 32.0 g/mL, with a limit of detection of 0.044 g/mL [134].

## 8. Cyanodegradation of Pesticides

Cyanoremediation utilizes cyanobacteria to remove, degrade, or transform pollutants such as heavy metals, dyes, and pesticides from wastewater and contaminated soils. Recent studies have proposed using low-cost algal systems to treat wastewater and agrochemical effluents commercially [137]. Various microalgae assimilate pesticides through biosorption and bioaccumulation depending on their lipid content, strain characteristics, and pesticide structure [138]. Microalgae can also utilize pesticides and cyanide as carbon and nitrogen sources [139]. Several cyanobacteria species have demonstrated the ability to bioremediate and degrade different categories of pesticides. Some reviews are available discussing in detail the utilization of cyanobacteria and microalgae for bioremediation of pesticides were published from time to time by Subramanian et al. [140], Kuritz [141], Jha and Mishra [142], Subashchandrabose et al. [143], Kumar and Singh [144], and Vijayan and Abdulhameed [145]. Recent findings and progress within the field under examination have been outlined in this section.

### 8.1. Insecticides

Endosulfan is a restricted-use insecticide effective against various agricultural pests, including aphids, fruit worms, beetles, leafhoppers, moth larvae, and whiteflies across multiple crop types. Two species of cyanobacteria, *Anabaena* sp. Strain PCC 7120 and *Anabaena flos-aquae*, were shown to biotransform the pesticide endosulfan into its primary metabolite endodiol, with trace amounts of the secondary product endosulfan sulfate detected [146]. El-Bestawy et al. [147] investigated the potential degradation of the pesticide lindane (*γ*-hexachlorocyclohexane) from agricultural runoff by environmental cyanobacteria species. All tested cyanobacteria species demonstrated abilities to degrade lindane. Sarasa-Buisán et al. [148] identified probable homologues of the lin genes in *M. aeruginosa*, which are involved in the degradation of lindane in *Sphingobium japonicum* UT26S, utilizing in silico methods.

Sahu and Gothalwal [149] examined the effectiveness of the cyanobacterium *Nostoc* in the removal of dimethoate, an organophosphorus insecticide. The organism eliminated 0.040 mg dimethoate in 20 days (90%) from a medium containing 50 ppm dimethoate. Also, the depletion from the medium was biological and stable in a lab environment. Another organophosphorus insecticide, PY, is effectively accumulated and degraded by *N. muscorum* to a safe environmental product, as reported by Hamed et al. [74]. Chlorpyrifos is also an organophosphorus insecticide that is known to be degraded by cyanobacteria. Vijayan and Abdulhameed [145] screened cyanobacteria for chlorpyrifos biodegradation, and *Coleofasciculus chthonoplastes* demonstrated excellent chlorpyrifos degradation abilities. Abou Elatta et al. [150] assessed the ability of the cyanobacterial strains *Anabaena oryzae* and *N. muscorum* to degrade chlorpyrifos. Additionally, it was investigated how inoculating rice with cyanobacterial strains and chlorpyrifos might affect the plant's development and yield. After 12 days of incubation, cyanobacterial strains degraded all components of chlorpyrifos. Singh et al. [151] reported that the *Synechocystis* sp. PUPCCC 64 can degrade the pesticide chlorpyrifos. Other strains of cyanobacteria such as *A. oryzae*, *N. muscorum*, and *Spirulina platensis* are capable of degrading and utilizing malathion as a phosphorus source. When grown under high concentrations of malathion, these strains exhibited increased biomass as well as elevated protein and carbohydrate content [152]. Tiwari et al. [153] isolated a paddy field cyanobacteria *Fischerella* sp. that can degrade and utilize the organophosphorus pesticide methyl parathion (MP) as a phosphate source. The same group also identified a new isolate of the genus *Scytonema* efficient in the degradation of MP [154]. Carbofuran is a carbamate insecticide widely used to control insects and nematodes on a variety of agricultural crops. Afify et al. [155] evaluated the biodegradation of the carbofuran by the survival of cyanobacterial strains *A. oryzae* and *N. muscorum* in the rice fields. The data showed the ability of cyanobacteria to degrade all components of carbofuran and the mixture of *N. muscorum* and *A. oryzae* had improved in dry weight and increased the nitrogen fixation in the environment.

Bano et al. [156] assessed the effectiveness of removing pyrethroids in two marine filamentous cyanobacteria, *Pseudanabaena* sp. and *Leptolyngbya* sp. Both strains were found to be effective at removing pyrethroids. In comparison to *Leptolyngbya* sp., which demonstrated removal effectiveness of 82.0% and 97.8% for cypermethrin and deltamethrin, *Pseudanabaena* sp. was shown to be more effective, removing up to 98.0% and 99.0% of cypermethrin and deltamethrin, respectively.

Sulfoxaflor (SFX) is a systemic neurotoxic insecticide that kills insects through contact or ingestion. Łukaszewicz et al. [157] assessed the potential of two common bloom-forming cyanobacteria species, *Synechocystis salina* and *M. aeruginosa*, to metabolize SFX.

### 8.2. Herbicides

Glyphosate is a broad-spectrum systemic herbicide. Lipok et al. [158] conducted an experiment demonstrating that a mixed culture of *Spirulina* sp. showed a notable capacity to degrade glyphosate in an aqueous environment. Indeed, Lipok et al. [159] reconfirmed that *S. platensis* and *Streptomyces lusitanus* catalyze glyphosate metabolism. Four cyanobacterial strains (*Anabaena* sp., *M. aeruginosa*, *Lentinula boryana*, and *Nostoc punctiforme*) utilized glyphosate as the sole phosphorus source [160]. Dyhrman et al. [161] reported that the marine cyanobacterium *Trichodesmium erythraeum* performs phosphorus-dependent glyphosate transformation. Forlani et al. [160] postulated that extracellular phosphatases are unlikely to substantially contribute to glyphosate degradation at large scales. Cyanobacterial strains possessing the ability to utilize this phosphonate as a phosphorus source have practical significance, as such strains could effectively remediate pesticides [162]. 2,4-D is reported to be degraded by *N. muscorum* Meg 1 to the byproduct 2,4-DCP. However, 2,4-DCP was also reported to be toxic and had a negative impact on these beneficial microorganisms [163]. Agrawal et al. [164] reported a novel protein aldo-keto reductase (AKR17A1) from *Anabaena* sp. PCC 7120 capable of degrading the rice field herbicide butachlor from the acetanilide class.

## 9. Consortia of Cyanobacteria/Microalgae and Bacteria for Pesticide Biodegradation: Biotechnological Potential

Microalgae and cyanobacteria have been shown to form symbiotic relationships with various aerobic and anaerobic microorganisms, associating together in community-defined consortia. Within these consortia, algal and bacterial groups interact synergistically, demonstrating enhanced capability for the biodegradation of organic and inorganic pollutants beyond the individual microorganism species alone. Through symbiotic interactions between constituent microorganisms, the collective algal-bacterial consortium is able to break down pollutants more efficiently than could be achieved by any single microbe operating independently [165]. In recent years, the use of bacto-microalgal consortia has gained popularity due to its excellent efficacy in the removal of pesticides. However, studies on bacterial consortia are more numerous.

Abdel-Razek et al. [166] analyzed pesticide degradation using a consortium of microalgae (*Chlorella vulgaris*, *Scenedesmus quadricauda*, and *S. platensis*) to remove the organophosphate pesticide malathion and heavy metals Cd, Ni, and Pb from water samples in Egypt containing combinations of urban wastewater and agricultural drainage water. The consortium demonstrated the potential for bioremediation of malathion and heavy metals from contaminated aqueous environments. Similarly, Cheng et al. [167] constructed an algae-bacteria system with municipal wastewater and analyzed the removal effects of the consortium system on IMI, a systemic insecticide, and conventional nutrients from municipal wastewater. The consortium efficiently removed IMI. Cheng et al. [168] developed a microalgae-bacteria system with municipal wastewater and reported efficient removal by the consortium system of IMI/thiacloprid (THI) and conventional nutrients. Kumari et al. [169] demonstrated artificial bacterial-algal consortia's potential for degrading lindane and organochlorinated pesticides in contaminated environments. Through integrated chemical analysis, they showed the consortia efficiently degraded and detoxified these compounds.

## 10. Conclusion and Future Prospects

Food production in huge amounts has turned into a challenge for agronomic trades due to the exponential growth in population. In order to solve this issue, industries have started relying on chemical pesticides and fertilizers, which not only pose a risk to human health but also have a detrimental impact on soil fertility, organic component depletion, affect crop quality, and lead to soil and water contamination. Pesticide residues have definite effects on human life; hence, it is crucial to comprehend their fate and behavior in the environment. In order to achieve high crop output, the use of pesticides and fertilizers containing high quantities of nitrogen has become essential in modern rice farming. Adopting practical and sustainable farming practices is therefore urgently needed to produce enough food at a reasonable cost for the world's expanding population. The potential of using cyanobacteria as a biological tool has received a lot of attention recently since they are essential for maintaining the fertility and integrity of soil, plant health, improving agricultural output, and reducing plant diseases. Exploration and the use of cyanobacteria-based biofertilizers will not only provide economic benefits but also protect our environment, human health, and the sustainability of natural ecosystems. The use of cyanobacterial biostimulants in agriculture could increase output from a specific area of land while decreasing reliance on synthetic fertilizers and promoting the transition to a sustainable intensification of agriculture. To prevent exposure to pesticides, policymakers might educate farmers about the benefits of using integrated pest management systems and good agricultural practices. The current paper represents an initial attempt to explore the toxicity of pesticides, the amount of pesticide residues, and their impact on Indian rice fields, along with sustainable solutions to get rid of them. Persistent organic pesticides have contaminated India; residues are present above MRL values in the soil, water, and air throughout the nation. Most studies reviewed indicate pesticide residue levels in Indian air, water, and soil are elevated despite low usage in India. This may be due to the fact that numerous chemical pesticides were widely utilized in the past before the majority of them were prohibited. An additional aspect contributing to residue levels in the environment of India is the application of pesticides at improper dosage, which results in an excess and off-target quantity. Additionally, there is a need to raise awareness among farmers about the potential benefits of cyanobacterial biofertilizers, and “organic farming” can become a reality in the future. The right pesticide application technology would reduce such waste and be more cost-effective. Using certified sprayers and nozzles should also be made known to farmers through education and training campaigns. Furthermore, the development of bacto-microalgal consortia should be encouraged as reports are still fragmentary as compared to bacterial consortia. This review provides an ideal approach for monitoring pesticide residues in rice and its environment. To reduce pesticide contamination, biopesticides should be developed alongside chemical pesticides. Further research is required to determine the application of appropriate processing technology to retain the grain's nutritional content while minimizing residue contamination. The bulk of acute pesticide poisoning, unwanted health effects, and environmental harm cannot be avoided or reduced unless preventative measures are put in place and firmly enforced, which will lead to sustainability.

## Figures and Tables

**Table 1:** Major classification of pesticides as proposed by Drum [33].

**I. Classification based on pesticide function and targeted pest organism**		
**Pesticide**	**Target pests**	**Example**
Acaricides	Mites	Bifenazate
Algicides	Algae	Copper sulfate
Avicides	Birds	Avitrol
Bactericides	Bacteria	Copper complexes
Fungicides	Fungi	Paclobutrazol
Herbicides	Weeds	Pendimethalin
Lampricides	Larvae of lamprey	Trifluoromethyl
Larvicides	Larvae	Temefos
Molluscicides	Molluscs (snails)	Metaldehyde
Nematicides	Nematodes	Aldicarb
Ovicides	Eggs of insects and mites	Benzoxazin
Piscicides	Fishes	Rotenone
Silvicides	Woody vegetation	Tebuthiuron
Rodenticides	Mice and rodents	Sodium monofluoroacetate
Termiticides	Termites	Fipronil
Viricides	Viruses	Scytovirin

**II. Classification according to the mechanism of entry**		
**Pesticide**	**Mode of entry**	**Example**
Systemic	Absorbed by plant	Glyphosate
Nonsystemic	Contact	Paraquat
Stomach toxicant	Introduced into the body through oral consumption and absorption via the gastrointestinal tract. Intoxication ensues, ultimately resulting in fatal toxicity	Malathion
Repellant	Keep pests away due to bad taste/odour	
Fumigant	Vapor enters the tracheal system (respiratory) of pests via spiracles	

**III. Classification of pesticide based on chemical composition**		
**Pesticide**	**Chemistry/source**	**Example**
Natural		
Plant-based		Azadirachtin
Mineral oils		
Synthetic		
Inorganic		Copper sulfate
Organic		
a. Organochlorines	Organic compound attached to 5 or more chlorine	Lindane
b. Organophosphates	Derivatives of phosphoric acid	Parathion
c. Carbamates	Derivatives of carbamic acid	Carbofuran
d. Synthetic pyrethroids	Duplicates of natural pyrethrins	Cypermethrin

**Table 2:** Details of pesticides and their impact on cyanobacteria found in paddy fields.

**Chemical class**	**Pesticide**	**Trade name**	**Recommended dose**	**Cyanobacteria**	**Experimental dose (mg/L)**	**Impact on treated cyanobacteria**	**Reference**
*Insecticide*							
Pyrethroids	Cypermethrin	Ustad, Cyperhit	300 mL/acre	*Anabaena PCC 7120* and *Nostoc muscorum ATCC*	2 and 4 mg/L	Reduces pigment contents and oxidative stress markers; enhancement of antioxidant defense system	Tiwari et al. [70]
	Alpha-cypermethrin	Fastac	20–40 mg/m^2^	*Anabaena* sp. *NC-K1*	2.5, 3.5, 4.5, 6.5, and 9.5 mg/L	Cell growth inhibition; reduces Chl-a content and carotenoids; induces oxidative damage	Chanu et al. [71]
	Cyhalothrin	Karate	0.5 mL/L	*Calothrix* sp. *(GUEco 1001)*	20, 40, 80, and 160 ppm	Significant decrease in dry weight biomass, Chl-a, car, phycocyanin, and nitrogen content	Gupta and Baruah [72]
Carbamate	Carbofuran and carbaryl	Furadan and Sevin	5 kg/acre and 4 g/L, respectively	*Nostoc paludosum*	2.5, 5, 10, 20, 50, 100, 250, and 500 ppm	Dose-dependent decrease in growth and nitrogen fixation	Shinde [73]
Organophosphate	Pyridaphenthion	Compendium	10 gm/acre	*Anabaena laxa* and *Nostoc muscorum*	2.5, 5, 7.5, 10, 15, and 20 mg/L	Cell growth inhibition, reduces Chl-a content and photosynthetic enzymes; induces oxidative damage	Hamed et al. [74]
	Malathion	Cythion	250–350 mL/acre	*Fischerella muscicola NDUPC001*	50, 100, and 150 mg/L	Lesser inhibitory effect on cell growth, biomolecules, nitrate reductase, and glutamine synthetase	Mishra and Dwivedi [75]
				*Nostoc ellipsosporum NDUPC002*	5, 10, and 15 ppm	Significant decrease in growth, Chl-a, NR, and GS activity and decline in carbohydrate and increased total protein	Mishra et al. [76]
	Chlorpyrifos	Brodan	500–750 mL/acre	*Spirulina platensis*	10, 20, 40, 60, 80, and 100 mg/L	Cell growth inhibition; reduces Chl-a content and carotenoids; enhancement of SOD activity	Bhuvaneswari et al. [77]
	Phorate	Thimate	4.9–7.5 lbs/ha	*Nostoc ellipsosporum*, *Scytonema simplex*, *Tolypothrix tenuis*, and *Westiellopsis prolifica*	0.40 ± 0.016, 0.50 ± 0.025, 0.52 ± 0.02, and 0.80 ± 0.03, respectively	Inhibit the activity of nitrogenase, glutamine synthetase (GS), and isocitrate dehydrogenase (ICDH)	Debnath et al. [78]
	Dimethoate	Rogor	1–1.3 mL/L	*Nostoc paludosum*	2.5, 5, 10, 20, 50, 100, 250, and 500 ppm	Dose-dependent decrease in growth and nitrogen fixation	Shinde [73]
Organochlorine	Endosulfan	Thiodane	1–2 lbs/acre	*Nostoc ellipsosporum*, *Scytonema simplex*, *Tolypothrix tenuis*, and *Westiellopsis prolifica*	0.025 ± 0.001, 0.029 ± 0.001, 0.048 ± 0.002, and 0.05 ± 0.002, respectively	Inhibit the activity of nitrogenase, glutamine synthetase (GS), and isocitrate dehydrogenase (ICDH)	Debnath et al. [78]
			—	*Anabaena fertilissima*, *Aulosira fertilissima*, and *Westiellopsis prolifica*	6, 30, and 20 ppm, respectively	Qualitative and quantitative proteome alteration	Nirmal Kumar et al. [79]
		Endotaf	—	*Nostoc paludosum*	2.5, 5, 10, 20, 50, 100, 250, and 500 ppm	Dose-dependent decrease in growth and nitrogen fixation	Shinde [73]
Systemic and contact	Acetamiprid	Pride	30–40 gm/acre	*Synechocystis* sp.	0.05, 0.1, 0.5, and 1 mM	Adverse effect on the photosystem-II activity	Li et al. [80]

*Herbicide*							
Phenoxy acids	2,4-D	Weedar 64	300 mL/acre	*Nostoc muscorum Meg 1*	50, 75, 100, and 125 mg/L	Reduces the activity of RuBisCO, nitrogenase, glutamine synthetase, and isocitrate dehydrogenase enzyme; alteration of thylakoid membrane	Sachu et al. [81]
				*Anabaena fertilissima*, *Aulosira fertilissima*, and *Westiellopsis prolifica*	30, 40, and 60 ppm	Qualitative and quantitative proteome alteration	Nirmal Kumar et al. [79]
Chloroacetamide	Butachlor	Machete	1000–1200 mL/acre	*Nostoc muscorum* and *Phormidium foveolarum*	5 and 10 mg/L	Cell growth inhibition; reduces photosynthetic pigments; enhancement of SOD activity and lipid peroxidation	Sheeba et al. [82]
	Pretilachlor	Craze, Rifit	1–1.5 L/ha	*Anabaena doliolum*	2, 5, 7, 10, 20, and 40 *μ*g/mL	Dose-dependent decrease in growth, total pigment content, and photosynthetic efficiency	Kanda et al. [83]
Sulfonyl urea	Monosulfuron	Tetmosol	25–40 gm/200–250 L of water	*A. flos-aquae*	0.03, 0.3, 3, 30, and 300 nmol/L	Affect the growth rate; inhibitory effect on protein synthesis	Shen et al. [84]
				*A. flos-aquae*, *Anabaena azollae*, and *A. azotica*	0.014, 0.029, and 0.22 mg/L	Stimulatory effect on heterocyst frequency and nitrogenase activity unlike an inhibitory effect on photosynthesis	Shen and Luo [85]
Triazine	Atrazine	Aatrex	600 gm/acre	*Cylindrospermum stagnale*	60, 80, 100, 120, and 140 mg/L	Reduces MDA content; enhancement of Chl content, total protein, and antioxidant enzyme activity	Ahmad et al. [86]
	Terbutryn	Igran	550–850 mL/ha	*Cyanobacterial mats*	0.031 *μ*mole L^−1^	Growth inhibition	El-Nahhal et al. [87]
Quaternary ammonium compounds	Paraquat	Gramoxone	1–1.5 L/acre	*Anabaena 7120*, *A. doliolum*, and *Anabaena L-31*	0.001, 0.002, 0.005, and 0.007 mg/L	Cell growth inhibition; induces oxidative damage; alternation in protein content	Panda et al. [88]
	Diquat	Reglone, Reglox, and Alligare	0.5–2.0 gallons/acre	*Cyanobacterial mats*	0.381 *μ*mole L^−1^	Growth inhibition	El-Nahhal et al. [87]
Organophosphorus	Glyphosate	Roundup	2 mL/L	*Microcystis aeruginosa*	1, 2, 5, and 10 mg/L	Increase in MDA, SOD, CAT, and peroxidase activity along with induced apoptosis and toxin release	Wu et al. [89]
				*Cylindrospermum indicum*, *Nostoc commune*, *N. linckia*, *Anabaena variabilis*, *Aulosira fertilissima*, and *Calothrix marchica*	100, 200, and 400 ppm	Lower concentrations of glyphosate were stimulatory, whereas higher concentrations were inhibitory. *Cylindrospermum indicum* was more sensitive to glyphosate, but *Aulosira fertilissima* and *Calothrix marchica* were the most tolerant	Bodkhe and Tarar [90]
Substituted urea	Diuron	Herbirex and Direx	500 g/ha	*Cyanobacterial mats*	0.009 *μ*mole L^−1^	Growth inhibition	El-Nahhal et al. [87]
Aromatic ether	Diclofop	Titan-Ag	3.4 kg active ingredient per hectare	*Microcystis aeruginosa*	1, 2, and 5 mg/L	Induced ROS generation and increased MDA content, SOD activity, and toxin release	Ye et al. [91]
	Diclofop methyl and diclofop acid			*Microcystis aeruginosa*	0.5, 1, 2, 5 mg/L	Ultrastructure changes indicated variable toxicity modes among tested chemicals. Toxicity order was S-DA < R-DA < DM < DA	Ye et al. [91]

*Fungicide*							
Acylalanine	Metalaxyl	Ridomil	1400 gm/acre	*Anabaena laxa* and *Nostoc muscorum*	5, 10, 15, 20, 25, and 30 mg/L	Cell growth inhibition; reduces photosynthetic pigment content and photosynthesis-related enzymes (PEPC and RuBisCo**)**	Hamed et al. [92]
Dithiocarbamate	Mancozeb	Bendaco	1.5–2 kg/ha	*Nostoc ellipsosporum*, *Scytonema simplex*, *Tolypothrix tenuis*, and *Westiellopsis prolifica*	40.0 ± 1, 25.5 ± 0.5, 50.5 ± 0.5, and 72.2 ± 2.2, respectively	Reduces the activity of nitrogenase and glutamine synthetase	Debnath et al. [78]
	Bagalol	—	—	*Nostoc ellipsosporum*, *Scytonema simplex*, *Tolypothrix tenuis*, and *Westiellopsis prolifica*	0.025 ± 0.001, 0.03 ± 0.001, 0.04 ± 0.002, and 0.042 ± 0.002, respectively	Reduces the activity of nitrogenase and glutamine synthetase	Debnath et al. [78]
Phenylurea	Pencycuron	Monceren	2.0 kg/tons of seed tubers	*Anabaena fertilissima*, *Aulosira fertilissima*, and *Westiellopsis prolifica*	30, 30, and 100 ppm, respectively	Qualitative and quantitative proteome alteration	Nirmal Kumar et al. [79]
Triazole	Tebuconazole	Folicur	500 L/ha	*Anabaena fertilissima*, *Aulosira fertilissima*, and *Westiellopsis prolifica*	15, 30, and 30 ppm, respectively	Qualitative and quantitative proteome alteration	Nirmal Kumar et al. [79]

**Table 3:** Pesticide residues observed in different parts of rice plant.

**S. no.**	**Pesticide**	**Rice agriculture**	**Residues detected**	**Half-life (days)**	**Reference**
1.	Pretilachlor	Rice grain and soil Irrigation water	< 0.001–0.05 *μ*g/g < 0.001–0.05 *μ*g/L	3.0–3.6	Arora et al. [131]

2.	Metamifop	Rice plant	0.02 ppm	3.5–2.2, 1.3–2.3, and 11.7–20.2	Saha et al. [132]

3.	Bispyribac sodium	Rice plant Rice hull Water	5.0 *μ*g/kg 2.0 *μ*g/kg 0.1 *μ*g/kg	3.0–3.8 5.0–5.6 soil 1.4–2.2	Zhang et al. [116]

4.	Pendimethalin	Grain of direct-seeded rice (DSR) and transplanted rice (TPR) Rice straw	0.005 and 0.007 *μ*g/g 0.003 and 0.005 *μ*g/g 0.003–0.006 *μ*g/g	23.51–24.66	Makkar et al. [121]

5.	DDTs and HCHs	Soil	3.5 *μ*g/kg	45	Tan et al. [130]

6.	Atrazine	Water and soil	0.01–1.7 *μ*g/kg	1.73	Qu et al. [133]

7.	Butachlor	Water and soil	0.044 g/mL	1.65–2.48 2.67–5.33	Puthalapattu et al. [134]

## Data Availability

The data that support the findings of this study are available from the corresponding authors upon reasonable request.
